# SHP2, SOCS3 and PIAS3 Expression Patterns in Medulloblastomas: Relevance to STAT3 Activation and Resveratrol-Suppressed STAT3 Signaling

**DOI:** 10.3390/nu9010003

**Published:** 2016-12-27

**Authors:** Cong Li, Hong Li, Peng Zhang, Li-Jun Yu, Tian-Miao Huang, Xue Song, Qing-You Kong, Jian-Li Dong, Pei-Nan Li, Jia Liu

**Affiliations:** 1 Liaoning Laboratory of Cancer Genetics and Epigenetics and Department of Cell Biology, Dalian Medical University, Dalian 116044, China; goodluck_licong@163.com (C.L.); lihongmcn@dlmedu.edu.cn (H.L.); zhangpenggirl821@sina.com (P.Z.); yulijundl1963@163.com (L.-J.Y.); huangtianmiao6688@aliyun.com (T.-M.H.); songxue0214@163.com (X.S.); kqydl@sina.com (Q.-Y.K.); 2 Department of Orthopedic Surgery, Second Hospital of Dalian Medical University, Dalian 116011, China; jldongdl@aliyun.com (J.-L.D.); delaho@126.com (P.-N.L.)

**Keywords:** medulloblastoma, STAT3 signaling, STAT3 negative regulators, PIAS3, resveratrol

## Abstract

Background: Activated STAT3 signaling is critical for human medulloblastoma cells. SHP2, SOCS3 and PIAS3 are known as the negative regulators of STAT3 signaling, while their relevance to frequent STAT3 activation in medulloblastomas remains unknown. Methods: Tissue microarrays were constructed with 17 tumor-surrounding noncancerous brain tissues and 61 cases of the classic medulloblastomas, 44 the large-cell medulloblastomas, and 15 nodular medulloblastomas, which were used for immunohistochemical profiling of STAT3, SHP2, SOCS3 and PIAS3 expression patterns and the frequencies of STAT3 nuclear translocation. Three human medulloblastoma cell lines (Daoy, UW228-2 and UW228-3) were cultured with and without 100 μM resveratrol supplementation. The influences of resveratrol in SHP2, SOCS3 and PIAS3 expression and SOCS3 knockdown in STAT3 activation were analyzed using multiple experimental approaches. Results: SHP2, SOCS3 and PIAS3 levels are reduced in medulloblastomas in vivo and in vitro, of which PIAS3 downregulation is more reversely correlated with STAT3 activation. In resveratrol-suppressed medulloblastoma cells with STAT3 downregulation and decreased incidence of STAT3 nuclear translocation, PIAS3 is upregulated, the SHP2 level remains unchanged and SOCS3 is downregulated. SOCS3 proteins are accumulated in the distal ends of axon-like processes of resveratrol-differentiated medulloblastoma cells. Knockdown of SOCS3 expression by siRNA neither influences cell proliferation nor STAT3 activation or resveratrol sensitivity but inhibits resveratrol-induced axon-like process formation. Conclusion: Our results suggest that (1) the overall reduction of SHP2, SOCS3 and PIAS3 in medulloblastoma tissues and cell lines; (2) the more inverse relevance of PIAS3 expression with STAT3 activation; (3) the favorable prognostic values of PIAS3 for medulloblastomas and (4) the involvement of SOCS3 in resveratrol-promoted axon regeneration of medulloblastoma cells.

## 1. Introduction

Medulloblastoma is the most frequent primary brain malignancy in childhood and is characterized by rapid and aggressive intracranial growth and high recurrence incidence [1]. Although the combination of operation with adjuvant radiotherapy and/or chemotherapy has been adapted in clinical settings [2], the outcome of medulloblastomas remains poor due to the difficulty in removing the highly aggressive tumor radically and the long-term side effects of conventional anticancer therapies [3,4]. It is therefore urgently necessary to investigate the critical molecular alterations related with medulloblastoma formation and progression and to explore more effective therapeutic approaches with lesser toxicities for better management of medulloblastomas.

Several signaling pathways are known to be involved in the formation and progression of medulloblastomas [5,6,7], of which STAT3 signaling seems most crucial because selective inhibition of STAT3 activation suppresses growth and induces apoptosis of medulloblastoma cells [5,8,9]. However, the underlying mechanism by which STAT3 signaling is inhibited by resveratrol remains largely unknown. It has been found that several factors can negatively regulate STAT3 signal transduction. For instance, induction of SHP-1 and SHP-2 tyrosine phosphatases inhibit constitutive and inducible STAT3 activation and loss of protein tyrosine phosphatase leads to aberrant STAT3 activation and promotes gliomagenesis [10,11]. Similarly, PIAS3 down-regulation is associated with increased STAT3 activation and poor prognosis of malignant mesothelioma patients [12]. In some types of human malignancies, an interplay between STAT3 signaling and SOCS3 has been found in the form of feedback control [13,14]. Nevertheless, no comprehensive study is so far available concerning the statuses of those negative regulators in medulloblastoma tissues and their relevance with STAT3 activation.

Resveratrol (3,5,4′-trihydroxy-trans-stilbene), a naturally occurring polyphenol found in grapes, peanuts and the root of polygonum cuspidatum, has preventive and therapeutic effects on many kinds of human cancers including brain malignancies [15]. More importantly, the in vitro and in vivo anticancer doses of resveratrol have little toxic effect on the normal tissues and cells [16,17,18]. For example, resveratrol in 100 μM is sufficient to cause growth arrest and apoptosis of human medulloblastoma and glioblastoma cells in vitro [16,19] and rat orthotopic glioblastomas in vivo without affecting glial cells and neurons [18]. These findings thus suggest that resveratrol would be of potential practical value in improving the therapeutic outcome of medulloblastomas. Our previous studies demonstrate that STAT3 signaling is the critical molecular target of resveratrol although other signaling pathways are inhibited concurrently in resveratrol-treated medulloblastoma cells [19,20,21]. However, the reasons for resveratrol-caused STAT3 inactivation remain to be clarified. The current study aims to address these issues using medulloblastoma microarrays to profile SHP2, SOCS3 and PIAS3 expression patterns in medulloblastoma tissues and resveratrol-sensitive medulloblastoma cell lines to elucidate the impact(s) of resveratrol in SHP2, SOCS3 and PIAS3 expression when exerting its inhibitory effect on STAT3 signaling and cell proliferation.

## 2. Experimental Section

### 2.1. Medulloblastoma Specimens and Microarray Construction

The protocol of this study had been reviewed by the Ethics Committee of Dalian Medical University before conducting the experiments. The archived 120 paraffin-embedded medulloblastoma specimens were kindly provided by the Clinical Pathology Departments, the First Affiliated Hospital of Dalian Medical University and Shen-Jing Hospital of China Medical University at Shenyang. This study was approved by the hospital institution review board and the informed consent was obtained from all patients before tissue sample collection. The tissue microarrays were constructed in duplicate with 120 medulloblastoma and, where possible, the noncancerous tumor-surrounding brain tissue blocks by the method described previously [22].

### 2.2. Tissue Microarray-Based Immunohistochemical Staining

The expression levels and intracellular distribution patterns of STAT3, SHP2, SCOS1, SOCS3 and PIAS3 in the three subtypes (the classical, the large-cell and the nodular) of medulloblastomas were profiled immunohistochemically, using paraffin sections of the constructed medulloblastoma microarrays. The antibodies used were the rabbit anti-human p-STAT3 (Proteintech, Chicago, IL, USA), SCOS1, SCOS3, PIAS3 and SHP2 (Santa Cruz Biotechnology, Inc., Santa Cruz, CA, USA) antibodies at dilutions of 1:120, 1:100, 1:100, 1:120 and 1:100, respectively. Color reaction was developed using 3, 3′-diaminobenzidine tetrahydrochloride (DAB). The sections without the first antibody incubation were used as the background control. According to the labeling intensity, the staining results were evaluated by two researchers, and scored as negative (−) if no immunolabeling was observed in target cells, weakly positive (+) if the labeling was faint, moderately positive (++) if the labeling was stronger, and strongly positive (+++) if the labeling was distinctly stronger than (++).

### 2.3. Cell Culture and Resveratrol Treatments

Human medulloblastoma UW228-2 and UW228-3 cell lines were kindly provided by Dr. Keles GE, Department of Neurosurgery, Washington University at Seattle. Human medulloblastoma DAOY cell line was obtained from the Cell Culture Facility, Chinese Academy of Sciences Cell Bank, Shanghai. The three cell lines were cultured in DMEM (Gibco Life Science, Grand Island, NY, USA) supplemented with 10% fetal bovine serum (Gibco, Grand Island, NY, USA) under 37 °C and 5% CO_2_ condition and were plated onto culture dishes (Nunc A/S, Roskilde, Denmark) at a density of 5 × 10^4^/ml, and incubated for 24 h before further experiments. For paralleled H/E staining, Immunocytochemical (ICC) labeling and transferase-mediated deoxyuridine triphosphate-biotin nick end labeling TUNEL assay (Promega, Madison, WI, USA), dozens of cell-bearing coverslips were concurrently prepared under the exact same experimental conditions using Nest-Dishes (Nest Biotech., Inc., Wuxi, China; China invention patent No. ZL200610047607.0) and collected regularly during drug treatments. Resveratrol (Res; Sigma-Aldrich, St. Louis, MO, USA) was dissolved in dimethylsulfoxide (DMSO; Sigma-Aldrich, St. Louis, MO, USA) and diluted with culture medium to the optimal working concentration (100 μM) just before use. The cells were treated by 100 μM RA for 72 h, while the cells under normal culture condition and treated by 0.2% DMSO were used as normal and background controls, respectively. Cell numbers and viabilities were checked in 12 h intervals and the cell-bearing coverslips were fixed in cold acetone or 4% paraformaldehyde (pH 7.4) for morphological, immunocytochemical examinations and TUNEL assay. The experimental groups were set in triplicate and the experiments as well as the following examinations were repeated for three times to establish confidential conclusion.

### 2.4. Immunocytochemical Staining

Immunocytochemical (ICC) staining for p-STAT3, SOCS1, SOCS3, PIAS3 and SHP2 was performed on the coverslips of the three medulloblastoma cell lines collected from different experimental groups. The antibodies and their dilutions are the same as that used in immunohistochemical staining for tissue microarray sections. The cell-bearing coverslips without the first antibody incubation were used as the background control.

### 2.5. Protein Preparation and Western Blotting

Total cellular proteins were prepared from the cells under different culture conditions. The sample proteins (15 μg/lane) were separated by electrophoresis in 10% sodium dodecylsulfate–polyacrylamide gel electrophoresis and transferred to polyvinylidene difluoride membrane (Amersham, Buckinghamshire, UK). The membrane was blocked with 5% skimmed milk in TBS-T (10 mM Tris–Cl, pH 8.0, 150 mM NaCl and 0.5% Tween 20) at 4 °C overnight, rinsed three times with TBS-T and followed by 3 h incubation at room temperature with the first antibody, and 1 h incubation with HRP-conjugated anti-rabbit IgG (Zymed Lab Inc., San Francisco, CA, USA). The bound antibody was detected using the enhanced chemiluminescence system (Roche, Penzberg, Germany). After removing the labeling signal by incubation with stripping buffer (62.5 mM Tris–HCl, pH 6.7, 100 mM 2-mercaptoethanol, 2% SDS) at 55 °C for 30 min, the membrane was reprobed with other antibodies one-by-one until all of the parameters were examined.

### 2.6. RNA Isolation and RT-PCR

Total cellular RNAs of the experimental groups were extracted using Trizol solution (Life Technology, Grand Island, NY, USA). The sample RNAs were subjected to reverse transcription/RT and then polymerase chain reaction/PCR using the primers specific for STAT3, PTP, SOCS1, SOCS3 and PIAS3 according to producer’s protocols (Takara Inc., Dalian Branch, Dalian, China). The sequences of PCR primers for each of the gene transcripts were listed in Table 1. The PCR products were resolved on ethidium bromide-stained 1.5% agarose gel and photographed under UV illumination (UVP, LLC, Upland, CA, USA). β-actin products generated from the same RT solutions were used as quantitative control.

### 2.7. siRNA Knockdown of SOCS3 Expression

UW228-3 cells were selected to elucidate the impact(s) of SOCS3 down-regulation in cell growth, STAT3 activation and resveratrol sensitivity by SOCS3-specific siRNA transfection (siSOCS3-1: 5′-CCAAGAACCTGCGCATCCA-3′; siSOCS3-2: 5′-AGAGCCTATTACATCTACT-3′) [23]. The mock oligonucleotides (sense-50-UUCUCCGAACGUGUCACGUTT-30 and antisense-50-ACGUGACACGUUCGGAGA) and β-actin siRNAs (sense-50-UGAAGAUCAAGAUCAUUGCdTdT-30 and antisense-50-GCAAUGAUCUUGAUCUUCAdTdT-30) were used as negative and positive controls of transfection efficiency [24]. Those siRNAs were synthesized by Genepharma Company, Shanghai, China. Briefly, UW228-3 cells were conventionally cultured in 6-well plates to 60% to 70% confluence and then transfected with 0.3 nmol siRNA/well for 2 or 3 days using 4 mL X-tremeGENE siRNA transfection reagent according to manufacturer’s manual (Roche, Penzberg, Germany). After confirming the efficiency of SOCS3 inhibition by RT-PCR, the transfectants were incubated in the medium without or with 100 μM resveratrol for 72 h; afterward, the cells were examined by morphological staining, viable and nonviable cell counting, STAT3- and SOCS3-oriented immunolabeling. The results were compared with those obtained from the normally cultured cells and the cells treated by mock oligonucleotides.

### 2.8. Statistical Analyses

The results obtained from tissue microarray based immunohistochemical profiling were evaluated with the independent-samples *t*-test and ANOVA. Data were presented as mean ± standard deviation (SD) of separate experiments (*n* ≥ 10). When required, *p*-values are stated in the figure legends.

## 3. Results

### 3.1. Frequent STAT3 Activation in Medulloblastomas

**Figure 1:**
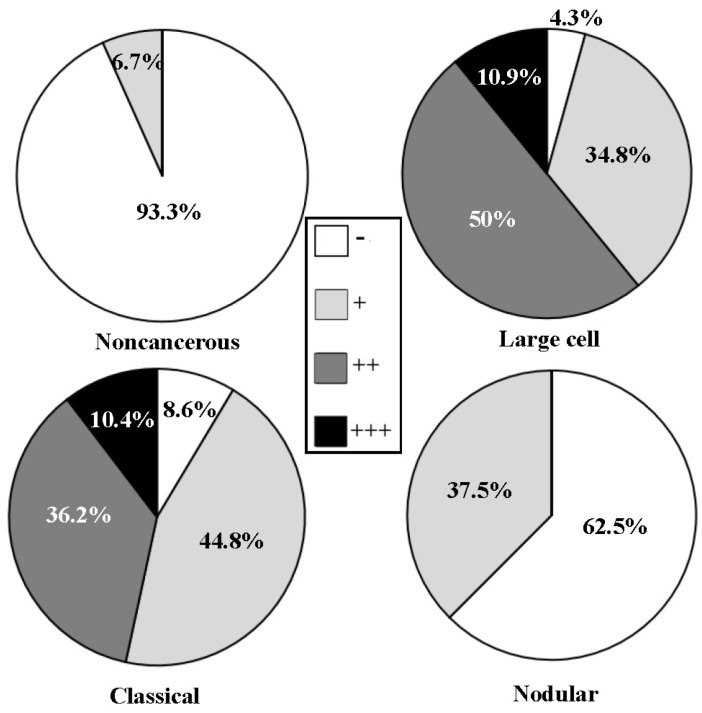
Incidences of p-STAT3 nuclear translocation in noncancerous brain tissues and the three histological subtypes of medulloblastomas.

According to the criteria of World Health Organization classification system [25], 120 medulloblastoma specimens were classified into three histological subtypes as classical (61 cases), large-cell (44 cases) and nodular (15 cases). The levels and intracellular distribution patterns of p-STAT3 in the three subtypes were analyzed according to the results of tissue microarray-based immunohistochemical staining. It was found that nuclear translocation of p-STAT3 could be observed in 63.9% (39/61) of the classical, 81.8% (36/44) of the large-cell, 53.3% (8/15) of the nodular medulloblastomas and 23.5% (4/17) of tumor-surrounding brain tissues (Figure 1). Statistical analyses (ANOVA) reveal that the staining densities and the frequencies of p-STAT3 nuclear translocation are significantly different between the three medulloblastoma subtypes and the tumor-surrounding brain tissues (*p* = 0.000).

### 3.2. Differential SHP2, SOCS3 and PIAS3 Expression Patterns

**Figure 2:**
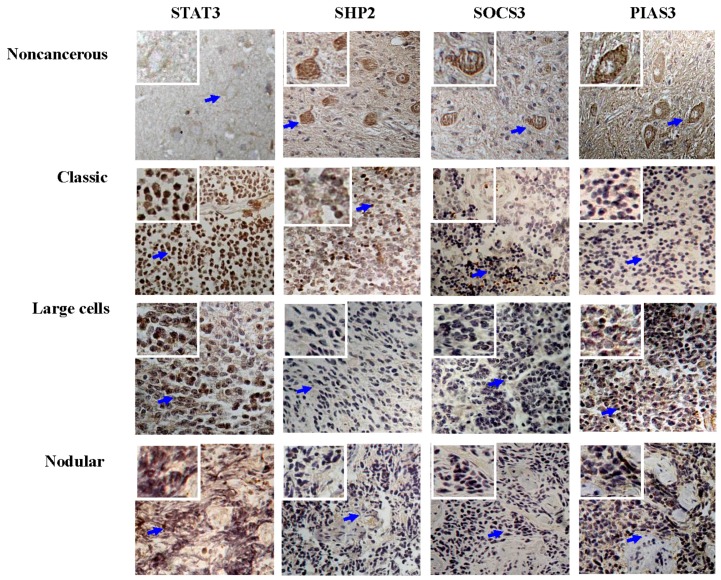
Immunohistochemical illustration (20×) of expression levels and intracellular distribution patterns of STAT3, SHP2, SOCS3 and PIAS3 in the three medulloblastoma subtypes and the tumor-surrounding cerebellum tissues. The arrows indicate the regions shown in the insets with higher magnification (40×).

**Figure 3:**
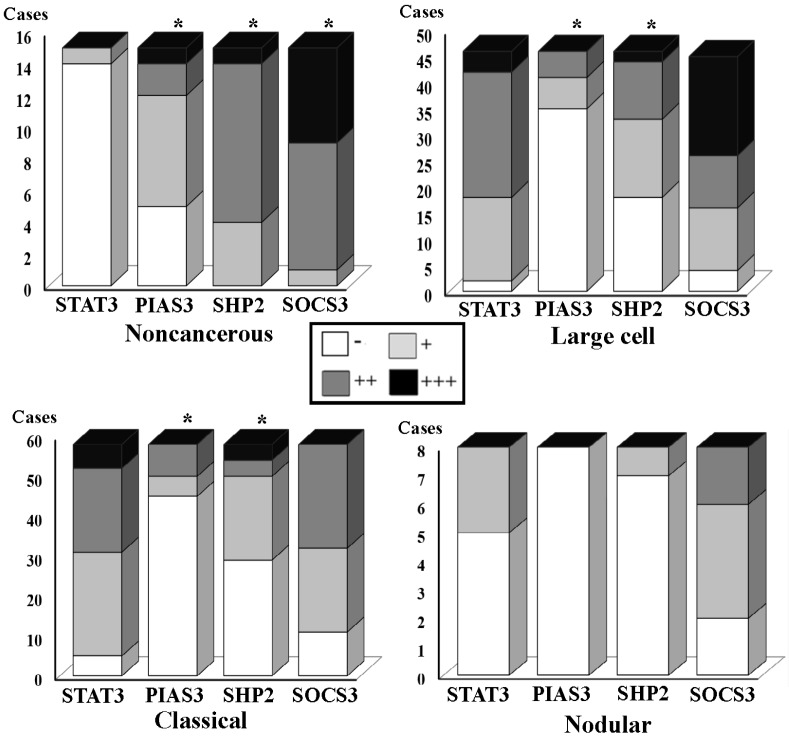
Fractionation of STAT3 nuclear translocation and SHP2, SOCS3 and PIAS3 expression levels in the tumor-surrounding noncancerous brain tissues (Upper left) and the large-cell (Upper right), classical (Low left) and nodular medulloblastomas (Low right). * Statistical analyses show significant reduction of their detection rates in comparison with that of the tumor-surrounding brain tissues (*p* = 0.000).

The results of immunohistochemical staining were summarized in Table 2 and shown in Figure 2 and Figure 3. It was revealed that the frequencies of p-SHP2 cytoplasmic labeling were significantly different between the tumor-surrounding brain tissues (17/17; 100%) and the classical (32/61; 52.5%; *p* = 0.016), the large-cell (27/44; 61.4%; *p* = 0.000) or the nodular medulloblastomas (2/15; 13.3%; *p* = 0.000). The frequencies of SOCS3 cytoplasmic detection were 100% (17/17) in the tumor-surrounding brain tissues, 80.3% (49/61) in the classical, 90.9% (40/44) in the large-cell and 80.0% (12/15) in the nodular medulloblastomas (Figure 3). Statistical analyses showed no significant differences between the tumor-surrounding brain tissues and the classical (*p* > 0.05), the large-cell (*p* > 0.05) or the nodular medulloblastomas (*p* > 0.05). In the case of PIAS3, significant differences of nuclear PIAS3 detection were evidenced between the tumor-surrounding brain tissues (58.8%; 10/17) and the classical (21.3%; 13/61; *p* = 0.000), the large-cell (15.9%; 7/44; *p* = 0.000) or the nodular medulloblastomas (6.70%; 1/15; *p* = 0.000).

### 3.3. STAT3 Activation and SOCS3 and PIAS3 Down-Regulation

The concurrent p-STAT3 nuclear translocation and p-SHP2, SOCS3 and/or PIAS3 down-regulation are summarized in Figure 3, followed by correlative analyses to elucidate the relevance of p-STAT3 nuclear translocation and the expression levels of p-SHP2, SOCS3 and PIAS3, respectively. Statistical correlations were established between p-STAT3 nuclear translocation and the level of SOCS3 (*R* = 0.333; *p* = 0.047) or PIAS3 expression (*R* = −0.494; *p* = 0.002) but not p-SHP2 down-regulation (*R* = 0.02; *p* > 0.05) in the large-cell medulloblastomas. Inverse correlation could be established between p-SHP2 (*R* = −0.35; *p* = 0.029), SOCS3 (*R* = 0.495; *p* = 0.001) or PIAS3 expression (*R* = −0.352; *p* = 0.020) and p-STAT3 nuclear translocation in the classic medulloblastomas. The corresponding data of the nodular group were not analyzed due to the limited case number.

### 3.4. Inhibited STAT3 Signaling in Resveratrol-Suppressed Cells

**Figure 4:**
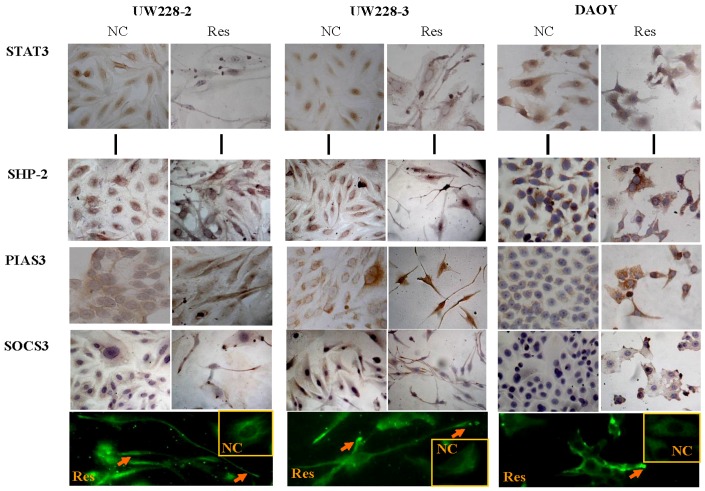
Immunocytochemical demonstration of STAT3, p-SHP2, SOCS3 and PIAS3 expression in three human medulloblastoma cell lines without (NC) and with 100 μM resveratrol treatment (Res) for 48 h (20×). Arrows in the immunofluorescent images (40×) indicate SOCS3 accumulation in the distal end of the axon-like processes of the three resveratrol-treated medulloblastoma cell lines. The inset images are the normally cultured cells.

**Figure 5:**
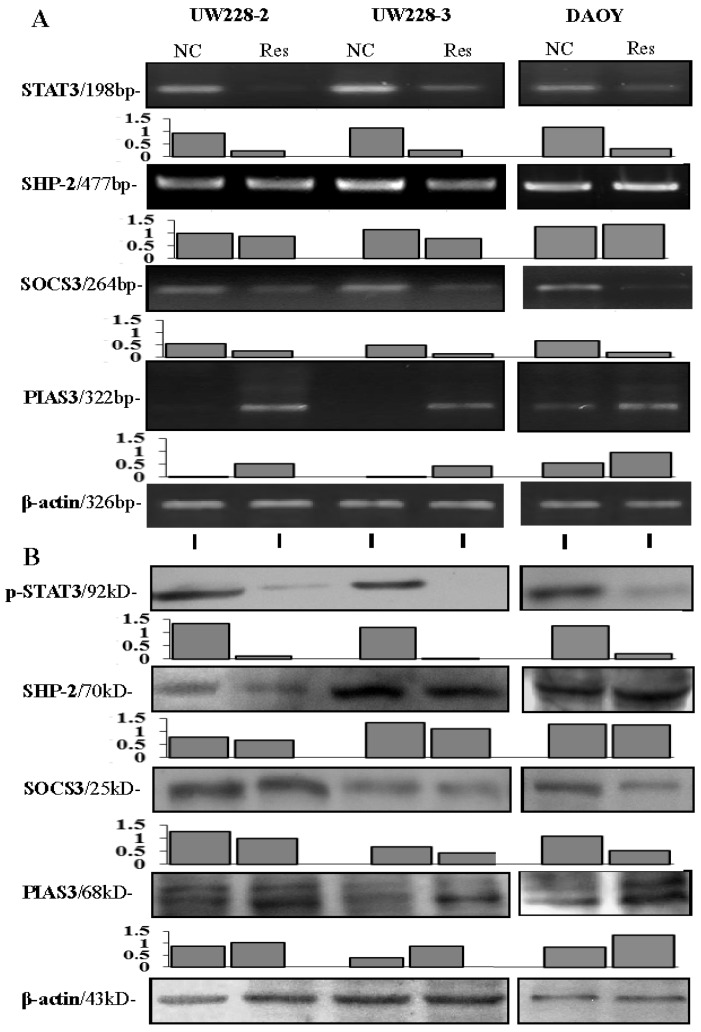
The results of RT-PCR (**A**) and Western blotting (**B**) and their grayscale analyses for STAT3, p-SHP2, SOCS3 and PIAS3 expression in three human medulloblastoma cell lines without and with 100 μM resveratrol treatment for 48 h.

Growth suppression, remarkable morphological alteration and frequent cell death were observed in UW228-2, UW228-3 and DAOY cells in a time-related fashion after 100 μM resveratrol treatment, and the majority of resveratrol-treated cells died of apoptosis at the 72-h time point [9,16]. As shown in Figure 4, high levels of STAT3 expression and distinct STAT3 nuclear translocation were observed in the three normally cultured medulloblastoma cell lines; the inhibitory effects of resveratrol on STAT3 signaling were evidenced in terms of reduction of STAT3 nuclear immunostaining (Figure 4) and down-regulated STAT3 expression (Figure 5).

### 3.5. Differential Responses of PIAS3, SOCS3 and SHP2 to Resveratrol 

The results of immunocytochemical staining (Figure 4), RT-PCR (Figure 5A) and Western blotting (Figure 5B) demonstrated that the level of PIAS3 was low in normally cultured UW228-2, UW228-3 and DAOY cells, which became increased with distinct nuclear labeling after resveratrol treatment for 48 h. SOCS3 levels were reduced in resveratrol-treated cells (Figure 5), accompanied with preferable accumulation of SOCS3 proteins in the distal end of the axon-like long process (Figure 4). SHP2 was expressed in the three cell lines and its level remained almost unchanged in resveratrol-treated cells.

### 3.6. SOCS3 Knockdown Caused Process Shortening

**Figure 6:**
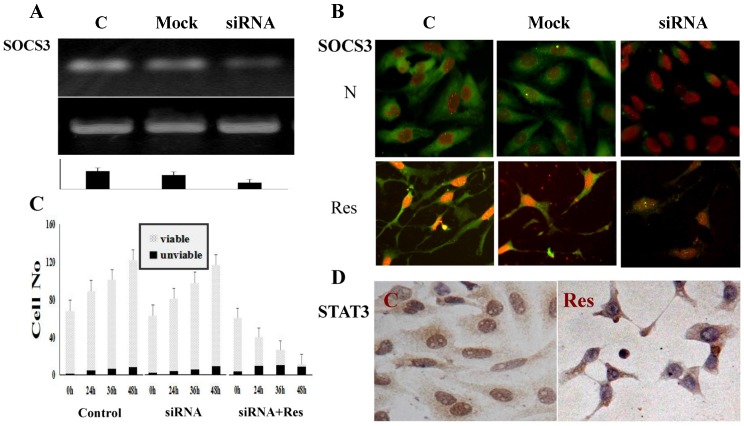
Knockdown of SOCS3 expression and its influence in STAT3 activation, resveratrol-sensitivity and axon-like process regeneration of medulloblastoma UW228-3 cells. (**A**) RT-PCR demonstration of SOCS3 downregulation in SOCS3-specific siRNA transfected UW228-3 cells (siRNA). The normally cultured (C), and mock RNA transfected (mock) cells are used as controls; (**B**) Immunofluorescent illustration (20×) of SOCS3 expression and intracellular distribution in normally cultured (C), mock RNA transfected (mock) and SOCS3-specific siRNA transfected UW228-3 cells (siRNA) before (N) and after resveratrol treatment (Res); (**C**) Viable:nonviable cell counting (mean cell number/visual field) reveals no influence of SOCS3 knockdown in the proliferation and resveratrol sensitivity of UW228-3 cells. N, UW228-3 cells without siRNA transfection; siRNA, normally cultured SOCS3 siRNA transfected UW228-3 cells; siRNA+Res, 100 μM resveratrol-treated UW228-3 cells with SOCS3 siRNA transfection; (**D**) SOCS3 knockdown exerts little effect on STAT3 activation in normally cultured cells (C) and resveratrol-caused STAT3 inactivation of UW228-3 cells (Res).

The results of RT-PCR demonstrate that the level of SOCS3 transcription is remarkably suppressed (Figure 6A), accompanied with decreased SOCS3 immuno-labeling in SOCS3 siRNA transfected cells (Figure 6B). H/E staining (data not shown) and viable:nonviable cell counting (Figure 6C) reveal that neither morphological alteration nor distinct cell death are found in the transfected population. p-STAT3-oriented immunocytochemical staining demonstrates p-STAT3 nuclear translocation in normally cultured transfectants and its remarkable reduction after resveratrol treatment (Figure 6D). After being treated by 100 μM resveratrol, the SOCS3 siRNA transfectants show distinct growth arrest in a time-related fashion (Figure 6C) without forming long axon-like processes (Figure 6B,D). SOCS3 level and growth of UW228-3 cells treated by 10 μM mock oligonucleotides are similar to those of normally cultured cells (Figure 6A,B).

## 4. Discussion

Severe short- and long-term adverse effects and frequent drug resistance are the major therapeutic dilemma in the management of childhood medulloblastomas [2,3,4]. It is therefore necessary to explore safer and more effective anti-medulloblastoma drugs. Our previous studies demonstrate that resveratrol efficiently suppresses growth and induces neuronal-like differentiation and extensive apoptosis of human medulloblastoma cells [9,19,20,21]. More importantly, this polyphenol compound has little harmful effect on rat glial cells and neurons in vitro and in vivo [16,18], suggesting its suitability for treating this sort of pediatric malignancy and the need to investigate the molecular event(s) occurring in resveratrol-treated medulloblastoma cells.

STAT3 signaling plays a pivotal role in regulating differentiation and proliferation of neuronal cells [26] and its activation is closely related with brain cancer formation [27,28]. Accordingly, our current study reveals that STAT3 nuclear translocation is frequently observed in classical and large-cell medulloblastomas in comparison with the tumor-surrounding brain tissues. According to the clinical data, the prognoses of medulloblastomas vary in a subtype-related manner. For instance, the patients with nodular tumors usually have relatively better prognoses, while the fates of the patients with classic and especially large-cell tumors are extremely poor [29]. Because some cancer prognostic factors such as VEGF, Bcl-2 and survivin are the downstream genes of STAT3 signaling, the constitutive STAT3 activation may lead to unfavorable outcomes for the large-cell and classic medulloblastomas. In this context, it would be worthwhile to elucidate the statuses of STAT3 regulatory factors and their correlations with STAT3 activation in medulloblastomas.

It has been recognized that STAT3 signaling can be regulated positively [28,29] or negatively [10,11,12,13,14]. Multiple factors have been identified as STAT3 signaling promoters, including IL-6 [30] and LIF [31] which are over expressed in medulloblastomas [32]. Nevertheless, we found that LIF was up-regulated in resveratrol-treated medulloblastoma cells with STAT3 inactivation presumably due to the feedback loop of STAT3 and LIF in those cells [9]. Alternatively, this finding suggests the presence of additional factor(s) that might negatively regulate STAT3 signaling. However, no report is so far available concerning the statuses of intrinsic STAT3 inhibitory factors in medulloblastomas and the impacts of resveratrol in their expression.

Several molecular factors are supposed to negatively regulate STAT3 signaling in different manners. PIAS3 inhibits STAT3 signaling by interrupting the interaction of p-STAT3 with its target genes [12]; SHP2 is a non-receptor type protein tyrosine phosphatases and its phosphorylated form (p-SHP2) interferes STAT-3 activation by dephosphorylating the active STAT-3 complexes in the cytoplasm and in nucleus [10]. SOCS3 works in a classic negative feedback loop to attenuate STAT3 activity by suppressive binding with phosphorylated JAK [14] and/or facilitating ubiqitination of JAK in the cytoplasm [33]. To shed light on the potential link(s) of these three negative regulators to STAT3 activation, their expression patterns in the three subtypes of medulloblastomas were profiled by tissue microarray-based immunohistochemical staining. The results revealed the general reductions of p-SHP2, SOCS3 and especially PIAS3 levels in the three medulloblastoma subtypes with frequent p-STAT3 nuclear translocation. It was also found that p-SHP2 downregulation was inversely correlated with STAT3 activation in classic but not large-cell medulloblastomas, suggesting differential regulation of STAT3 signaling in the histological subtypes of medulloblastomas. It would be therefore necessary to clarify whether the downregulation of those negative regulators is directly linked to or merely the paralleled molecular events with STAT3 activation. Alternatively, it would be more convincing if the levels of SHP2, SOCS1/SOCS3 and/or PIAS3 are altered accordingly in medulloblastoma cells with suppressed STAT3 signaling.

Our previous results clearly demonstrated that resveratrol can attenuate STAT3 activation of medulloblastoma cells via inhibiting STAT3 transcription and nuclear translocation [9]. Therefore, the resveratrol-treated medulloblastoma cells would be an ideal model to evaluate the role of SHP2, SOCS3 or PIAS3 in regulating STAT3 signaling. It is found that upon resveratrol treatment, PIAS3 is up-regulated and translocalized in nuclei, SOCS3 level is reduced and SHP2 remains unchanged in the three medulloblastoma cells so far checked. These findings thus suggest that of the three so-called STAT3 negative regulators examined here, PIAS3 rather than the other two proteins may be more correlated with resveratrol-caused STAT3 inactivation.

It has been proposed that SOCS3 is up-regulated as a feedback response to STAT3 activation [13,14]. Therefore, it would be possible that SOCS3 level is reduced in resveratrol-treated medulloblastoma cells with suppressed STAT3 signaling. The maintenance of STAT3 activation in SCOS3 siRNA transfected UW228-3 cells is in accordance with this notion. It has been evidenced that resveratrol inhibits growth and induces neuronal-like differentiation of human medulloblastoma cells [9]. Our finding of preferable accumulation and intracellular rearrangement of SOCS3 to the synapse-like end of the long processes indicates the involvement of this protein in axon regeneration [34]. This speculation is elucidated and proved by treating SOCS3 siRNA transfected UW228-3 cells with resveratrol, because the transfectants remain sensitive to resveratrol but fail to form axon-like long processes. These findings also suggest SOCS3 as a potential indicator of resveratrol-promoted neuronal differentiation of medulloblastoma cells.

## 5. Conclusions

Taken together, the expression patterns of three STAT3 negative regulators and their correlation with STAT3 activation in human medulloblastomas are investigated in this study. The results reveal that PIAS3 downregulation is more reversely correlated with STAT3 activation. PIAS3 upregulation and nuclear translocation in resveratrol-treated medulloblastoma cells with suppressed STAT3 signaling further suggests the negative regulatory effects of PIAS3 on STAT3 signaling and the potential value of PIAS3 in evaluating the prognosis of medulloblastoma patients. Inhibition of resveratrol-induced long process formation by SOCS3 siRNA transfection suggests the active role of SOCS3 in axon regeneration. In the future, we will further elucidate the influence of PIAS3 manipulation in the survival and resveratrol sensitivities of medulloblastoma cells.

## Figures and Tables

**Table 1:** Sequences of PCR primers for SHP-2, SOCS3, PIAS3, β-actin amplifications.

	Primer Sequences	Annealing Temperature	Product Size	Reference
SHP-2	F: 5′-CGAGTGATTGTCATGACAACG-3′	56 °C	477 bp	[16]
	R: 5′-TGCTTCTGTCTGGACCATCC-3′			
SOCS3	F: 5′-GAGCCCCCTCCTTCCCCTCGC-3′	56 °C	264 bp	[21]
	R: 5′-GGTCCAGGAACTCCCGAATG-3′			
PIAS3	F: 5′-ACGCTGTTGGCCCCTGGCAC-3′	56 °C	411 bp	[22]
	R: 5′-GGGGCTCGGCCCCATTCTTGG-3′			
β-actin	F: 5′-GCATGGAGTCCTGTGGCAT-3′	58 °C	326 bp	[17]
	R: 5′-CTAGAAGCATTTGGGGTGG-3′			

**Table 2:** p-STAT3, p-SHP2, SCOs3 and PIAS3 expression in three subtypes of medulloblastomas and cerebellum tissues.

	*n*	p-STAT3			*p*	p-SHP2			*p*	SOCS3			*p*	PIAS3			*p*
		−	+	≥++		−	+	≥++		−	+	≥++		−	+	≥++	
		(%)	(%)	(%)		(%)	(%)	(%)		(%)	(%)	(%)		(%)	(%)	(%)	
**Noncancerous**	15	14	1	0		0	4	11		0	1	14		5	7	3	
		(93.3)	(6.7)	(0.0)		(0.0)	(26.7)	(73.3)		(0.0	(6.7)	(93.3)		(33.3)	(46.7)	(20.0)	
**MB**	112	12	45	55	0.000 ^#^	54	36	22	0.000 ^#^	17	37	58	0.003 ^#^	88	11	13	0.001 ^#^
		(10.7)	(40.2)	(49.1)		(48.2)	(32.1)	(19.7)		(15.2)	(33.0)	(51.8)		(78.6)	(9.8)	(11.6)	
***Large***	46	2	16	28	0.000 *	18	15	13	0.001 *	4	12	30	0.000 *	35	6	5	0.000 *
		(4.3)	(34.8)	(60.9)		(39.1)	(32.6)	(28.3)		(8.7)	(26.1)	(65.2)		(76.1)	(13.0)	(10.9)	
***Classic***	58	5	26	27	0.000 ^&^	29	21	8	0.001 ^&^	11	21	26	0.004 ^&^	45	5	8	0.000 ^&^
		(8.6)	(44.8)	(46.6)		(50.0)	(36.2)	(13.8)		(19.0)	(36.2)	(44.8)		(77.6)	(8.6)	(13.8)	
***Nodular***	8	5	3	0		7	1	0		2	4	2		8	0	0	
		(62.5)	(37.5)	(0.0)		(87.5)	(12.5)	(0.0)		(25.0)	(75.0)	(25.0)		(100)	(0.0)	(0.0)	

^#^: Noncancerous vs. Large; *: Noncancerous vs. Classic; ^&^: Noncancerous vs. Nodular.
